# Morphological classification of neurons based on Sugeno fuzzy integration and multi-classifier fusion

**DOI:** 10.1038/s41598-024-66797-1

**Published:** 2024-07-11

**Authors:** Fuyun He, Guanglian Li, Haixing Song

**Affiliations:** 1 School of Electronic and Information Engineering, Guangxi Normal University, Guilin, 541004 China; 2 Guangxi Key Laboratory of Brain-inspired Computing and Intelligent Chips, Guangxi Normal University, Guilin, 541004 China

**Keywords:** Computational biology and bioinformatics, Classification and taxonomy, Computational neuroscience, Image processing, Machine learning

## Abstract

In order to extract more important morphological features of neuron images and achieve accurate classification of the neuron type, a method is proposed that uses Sugeno fuzzy integral integration of three optimized deep learning models, namely AlexNet, VGG11_bn, and ResNet-50. Firstly, using the pre-trained model of AlexNet and the output layer is fine-tuned to improve the model’s performance. Secondly, in the VGG11_bn network, Global Average Pooling (GAP) is adopted to replace the traditional fully connected layer to reduce the number of parameters. Additionally, the generalization ability of the model is improved by transfer learning. Thirdly, the SE(squeeze and excitation) module is added to the ResNet-50 variant ResNeXt-50 to adjust the channel weight and capture the key information of the input data. The GELU activation function is used to better fit the data distribution. Finally, Sugeno fuzzy integral is used to fuse the output of each model to get the final classification result. The experimental results showed that on the Img_raw, Img_resample and Img_XYalign dataset, the accuracy of 4-category classification reached 98.04%, 91.75% and 93.13%, respectively, and the accuracy of 12-category classification reached 97.82%, 85.68% and 87.60%, respectively. The proposed method has good classification performance in the morphological classification of neurons.

## Introduction

Neurons are the fundamental units of the brain, which constitutes the essential tissue of the nervous system. They transmit information through electrical and chemical signals, transmiting information from one neuron to another neuron or other type of cell. The transmission and processing of information is crucial to brain function. There are various forms and functions of neurons in the brain, which are connected together through complex networks to form complex neural circuits and functional networks^1–3^. By solving the neuronal classification problem, the characteristics of different neuronal types can be revealed, thereby helping researchers understand the pathogenesis of neurological diseases. In addition, it is also of great significant to explore the basic structure and function of the brain, human development, aging and other aspects of research, as well as related research in the fields of pathology and pharmacology^4–7^.

Deep learning is developing very rapidly in biomedical processing and has made many important advances^8,9^. However, the morphology of neurons is diverse and complex, and there are small morphological differences between different neurons such as the number, length and shape of branches, which increases the difficulty of classification. Besides, the ability of a single model to extract features is limited and cannot provide more comprehensive information to help with classification. Integration of multiple models based on ensemble learning can reduce the bias and variance of a single model classification^10^. However, traditional integration techniques, such as simple average method, random weight average and majority voting principle, usually assume that each model is independent and has the same weight, ignoring the differences among models, and the fixed integration and weight allocation methods lack flexibility^11^. Therefore, when dealing with neuron data of the same type but different subclasses, it is important not only to extract more neuron image features, but also to adopt appropriate integration methods to effectively fuse the features. The following main contributions of this research are as follows.

- In this paper, a method of multi-classifier fusion using Sugeno fuzzy integral is proposed for morphological classification of neurons.
- Before the model fusion, the three basic networks were optimized to improve the accuracy of classification.
- Experimental results of multi-classifier fusion on the NeuroMorpho-rat dataset show that this model achieves better classification accuracy than existing neuromorphic classification models.

Furthermore, the remaining structure and organization of the paper are as follows. In "Related work", the related work on morphological classification of neurons is introduced. In "Methodology", three improved network structures and the Sugeno fuzzy integration algorithm are introduced. "Experiments" discusses the experimental results and analysis. Finally, "Conclusions" summarizes the work accomplished.

## Related work

In recent years, remarkable progress has been made in the application of deep learning to neuron morphological classification, and the commonly employed models in this field include DenseNet^12^, GoogLeNet^13^, ResNet^14^, etc. In the classification of neuron morphology, the deep learning model can directly process the original morphological image data. By training large-scale neural networks, it can learn more distinctive feature representations. However, the structure of these models is too simple to fully account for the complexity of neuronal morphology, resulting in limited classification accuracy.

Alavi et al.^15^ performed two-dimensional and three-dimensional (3D) image analysis of neurons to obtain neuronal morphological information, and then classified dopaminergic neurons in the rodent brain stem. They compared the performance of three commonly used classification methods, including Support Vector Machines (SVM), Back Propagation Neural Networks (BPNNs), and Polynomial Logistic Regression. Han et al.^16^ proposed a new method for classifying neurons, which is based on the spatial structure of neurons. SVM was used to classify neurons, treating them as irregular fragments, and utilizing fractal geometry to describe their spatial structure. Evelyn et al.^17^ proposed a new neuronal morphological analysis method to characterize and classify neuronal cells by considering different levels of the dendritic tree. The method involves employing various strategies to dismantle the hierarchy of dendritic trees, enabling the identification of crucial components associated with the classification task, potentially linked to specific neuronal functions. Song et al.^18^ proposed a neuron model with dendrite morphology called the Logical Dendrite Neuron Model (LDNM) for neuron classification.

Lin et al.^19^ proposed two classification methods, one based on local accumulative connection deep neural network, the other based on complete accumulative deep neural network, and used the adaptive projection algorithm of neurons to project three-dimensional voxel coordinate points into two-dimensional, so as to obtain two-dimensional neuron images. Reference^20^ utilized full 3D neuronal voxel data for scaling and conducted experiments on neuron geometric morphology classification using a 3D convolutional neural network. The study by reference^21^ used naive Bayes and other machine learning methods to classify neurons on a dataset of 200 three-dimensional neurons. Zhang et al.^22^ proposed that different types of deep neural network modules can handle various types of features well, and built a Tree-RNN module for compressed but unstructured SWC format data. A convolutional neural network (CNN) module is constructed for 2D or 3D slice format data, and finally the features extracted from the two models are fused for final classification. Ofek et al.^23^ proposed a method for classifying neuronal cell types using local sparse networks, based on the study of electrophysiological activity in neurons.

These deep learning-based methods for classifying neuronal morphology play a crucial role in the field of brain neuroscience. However, these methods have not completely solved the problem of accurately classifying neuron morphology, and there is a need to improve the accuracy rate. This paper proposes a method that utilizes AlexNet^24^ , VGG11_bn^25^, and ResNet-50^14^ networks as neural network classifiers. The output results of these classifiers are then fused using the Sugeno fuzzy integral^26,27^. By synthesizing the output of multiple information sources, we can obtain more comprehensive and accurate results.

## Methodology

The AlexNet network uses the ReLU activation function and Dropout regularization to avoid the gradient disappearance problem and reduce the risk of overfitting. VGG11_bn model has good generalization ability by using small convolution kernel and maximum pooling layer. The advantage of ResNet-50 is the introduction of residual connections, making its network structure deeper and able to extract more advanced features, which can be applied to complex image classification tasks. All three network models have distinct advantages and significant differences, ensuring that their predictions on samples are not biased towards any specific class. As a result, for the classification of neuron morphology, AlexNet network, VGG11_bn network and ResNet50 network are selected as the basic network to construct the multi-classifier model.

**Figure 1:**
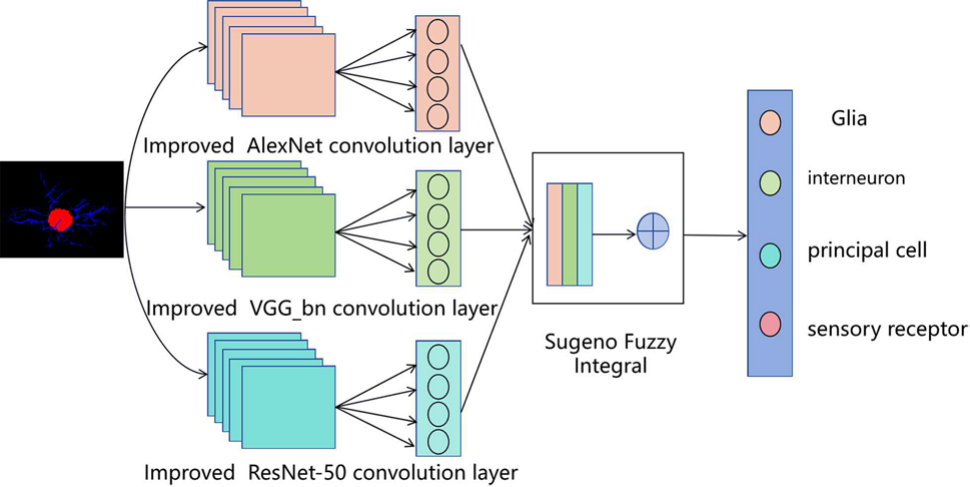
Overall structure of MCF-Net network.

In addition, in order to improve the classification performance of the model, each network is improved according to its own characteristics. For example, using AlexNet’s pre-trained model and fine-tuning its output layer to speed up the training process and improve model performance. In the VGG11_bn network, the GAP^28^ is used to replace the traditional fully connected layer to reduce the number of parameters. The SE^29^ module is introduced into ResNeXt-50^30^ to make it better extract neuron cell features, and the activation function is changed to GELU^31^ to help the model better adapt to changes in input data. The resulting Network is named Multiple Classifiers Fusion Network(MCF-Net). The overall structure of MCF-Net network is shown in Fig. 1. Firstly, neuron dataset images are input into the improved AlexNet, improved VGG11_bn and improved ResNet-50 networks for feature extraction, and classification prediction probabilities of each model are saved in a csv file. Then, the output results of each model are fused using Sugeno fuzzy integration algorithm. Finally, the classification output for the test set is obtained.

### Improved AlexNet

**Figure 2:**
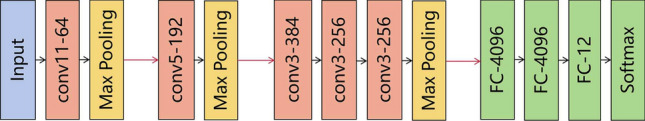
AlexNet network structure.

AlexNet model was proposed by Alex Krizhevsky, Ilya Sutskever and Geoffrey Hinton^24^. The model has 8 layers, of which 5 are convolutional layers and 3 are fully connected layers. This model has achieved breakthrough results in the ImageNet^32^ image classification challenge. The kernels of the second, fourth, and fifth convolution layers are only connected to the kernel maps in the previous layer located on the same GPU (as shown in Fig. 2). The kernels of the third convolution layer are connected to all the kernel maps in the second layer, and the neurons in the fully connected layer are connected to all the neurons in the previous layer.

Because of the simple structure of the AlexNet model, it has some limitations when applied directly to complex neurons.Therefore, AlexNet’s structure is modified to remove its two fully connected layers, leaving only one fully connected layer, and the weights of this new layer are randomly initialized. The convolutional layer weights in the pre-trained model are then used to initialize the modified network and fine-tune it to fit the new task. The AlexNet network structure is shown in Fig. 2.

### Improved VGG11_bn

The VGG11_bn model proposed by Karen Simonyan and Andrew Zisserman^25^, uses multiple 3$\times$3 convolutional layers stacked to increase the depth of the network. It also includes pooling layers for downsampling to reduce the size of the feature map while retaining important features. In addition, the VGG11_bn model adds a batch normalization layer between each convolutional layer and the fully connected layer, which can accelerate the convergence speed of the network and improve the stability and generalization ability of the model.

**Figure 3:**
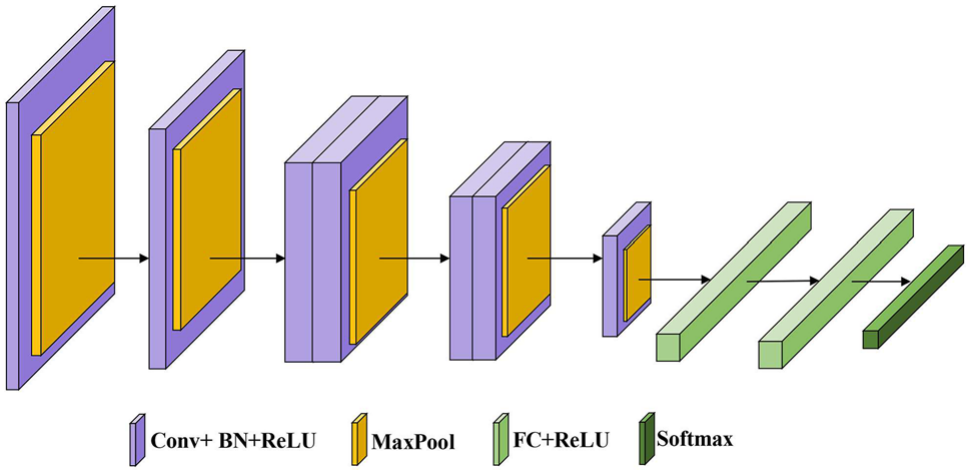
VGG11_bn network structure.

**Figure 4:**
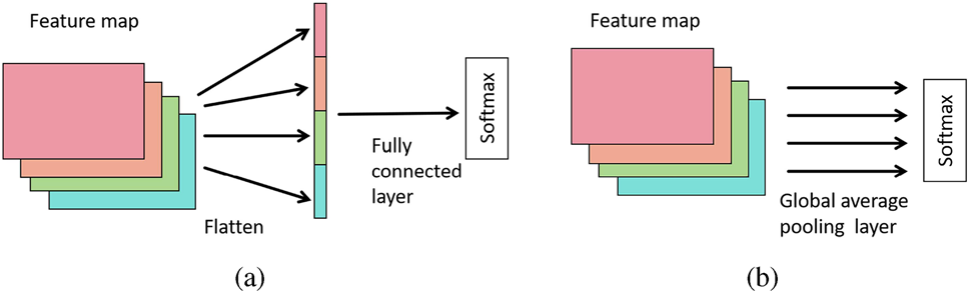
(**a**) Traditional fully connected layer, (**b**) global average pooling layer.

However, too large network training parameters are not conducive to model training, and the model structure is too deep, which is prone to gradient disappearance or gradient explosion. In order to solve the problem caused by excessive parameters, inspired by GAP and transfer learning^33^, the GAP is adopted to replace the traditional fully connected layer, and then a Softmax classification layer is connected and the output layer is fine-tuned, so a new training network structure is formed. Finally, transfer learning is performed on the weights pre-trained by VGG11_bn on ImageNet. Replacing the dense fully connected layer with GAP not only reduces model parameters but also prevents model overfitting. The VGG11_bn network is shown in Fig. 3. The traditional fully connected layer and the global average pooling layer are shown in Fig. 4.

### Improved ResNet-50

ResNet-50 has 50 layers including stacked convolution layers, pooling layers, fully connected layers and residual blocks. In the residual blocks, ResNet-50 uses 1$\times$1 convolution kernel for dimensionality reduction and expansion operations, which reduce the number of parameters and improving computational efficiency. After ResNet-50, researchers made some improvements to the model. For example, ResNeXt network combined residual network with Inception^34^ by introducing group convolution, which increased the diversity and expressiveness of the network.

**Figure 5:**
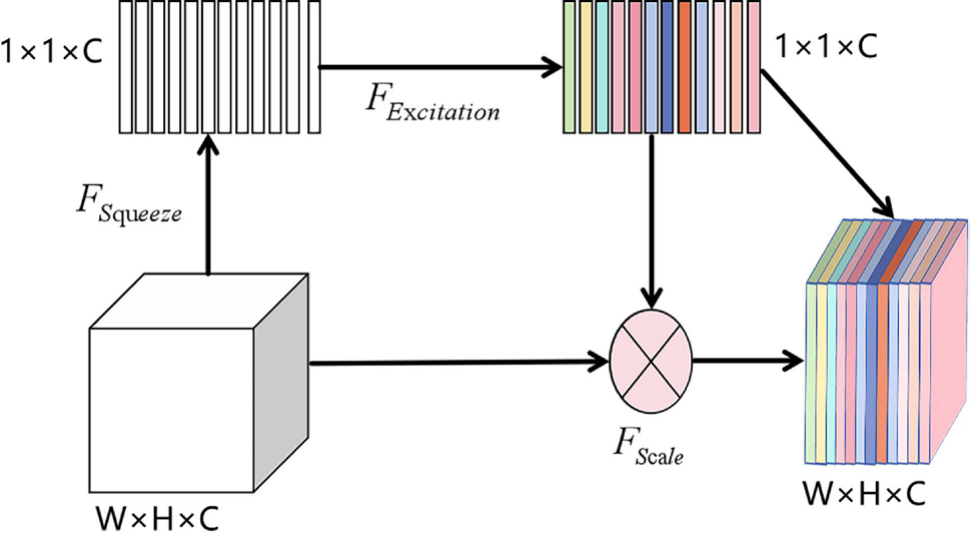
SE module structure.

**Figure 6:**
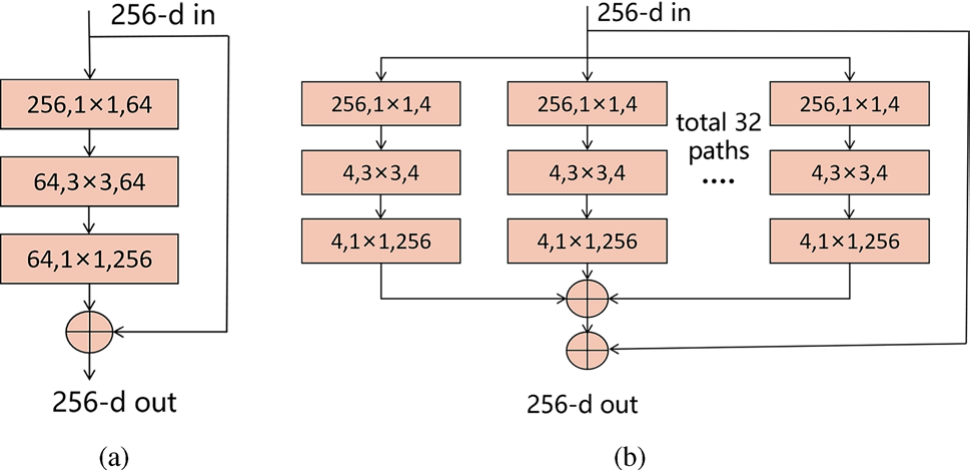
(**a**) ResNet-50 basic unit, (**b**) ResNeXt-50 residual structure.

In order to further optimize the ResNet-50 network model and improve the classification performance of the model, ResNeXt network is taken as a variant of ResNet-50, and SE-Net module is added to each basic unit of ResNeXt, so that the network can enhance the attention degree of important features, thus improving the expressiveness and accuracy of the model. SE module is shown in Fig. 5. Squeeze, Excitation and Scale operations are performed on the input feature map successively to obtain a feature map with channel weights. Relevant expressions are shown in Eqs. (1)–(3). In addition, GELU is used to replace the original RELU activation function, which helps improve the generalization ability of the model and made it easier to adapt to different data distributions. Figure 6 shows the residual structure of ResNet-50 and ResNeXt-50.

1 $$\begin{aligned} z_{c}= & {} F_{squeeze}(u_{c})=\frac{1}{H\times W }\sum _{i=1}^{H}\sum _{j=1}^{W}u_{c}(i,j) \end{aligned}$$

2 $$\begin{aligned} s= & {} F_{excitation}(z,W)=\sigma [W_{2}\sigma (W_{1}z)] \end{aligned}$$

3 $$\begin{aligned} X_{c}= & {} F_{scale}(u_{c},s_{c})=s_{c}u_{c} \end{aligned}$$

### Sugeno fuzzy integral

Sugeno fuzzy integral is a nonlinear integration method based on fuzzy set theory, which can effectively integrate multiple fuzzy sets to obtain a comprehensive and more representative output. Compared with other integrated technologies, it shows significant advantages in pattern recognition problems such as image classification^11,35^. Especially in dealing with ambiguity and uncertainty, Sugeno fuzzy integral can deal with noise, fuzzy boundary and missing information more flexibly by introducing fuzzy sets and membership functions. At the same time, it can also adapt to different data distributions and characteristics, capture and process nonlinear relationships to ensure the accuracy and reliability of classification results.

Let $X=\left\{ x_{1},...,x_{n} \right\}$ is a nonempty finite set with n elements, and *P*(*X*) is a power set of the set *X*. If the set function $\Omega$: $P(X)\rightarrow [0,1]$satisfies the following conditions:

1. $$\Omega (\Phi )=0,\Omega (X )=1$$
2. $$A\subset B\subset X$$, implies $$\Omega (A)\le \Omega (B)$$

Then $\Omega$ is a fuzzy measure defined on *X*^36–38^.

The Sugeno fuzzy-$\lambda$ measure was introduced by Tahani et al^39^. Let g be a fuzzy measure on the set $X=\left\{ x_{1},...,x_{n} \right\}$, then N is the number of information sources (in this case, N=3) and $A,B\in X$. The Sugeno-$\lambda$ measure is this function $f_{\lambda }:2^{X} \rightarrow [ 0, 1]$, and thus satisfies the following conditions:

1. $$f_{\lambda }(X)=1$$
2. If $A\cap B=\Phi$, then $\exists \lambda > -1$, such that, Expression (4) holds true.

4 $$\begin{aligned} g(A\cup B)=g(A)+g(B)+\lambda g(A)g(B) \end{aligned}$$

Where $\lambda \ge 1$ and $\lambda \ne 0$, then the fuzzy measure g is $g_{\lambda }$. When g is a $g_{\lambda }$ fuzzy measure, the fuzzy measure value $g(\left\{ x_{i} \right\} )$ of the singleton set $\left\{ x_{i} \right\} (i=1,2,...n)$is also referred to as the fuzzy density^35,40^.

$\lambda$ value in $g_{\lambda }$ fuzzy measure can be obtained by expression (5).

5 $$\begin{aligned} \lambda +1=\prod _{i=1}^{n}(1+\lambda g_{i}) \end{aligned}$$

**Figure 7:**
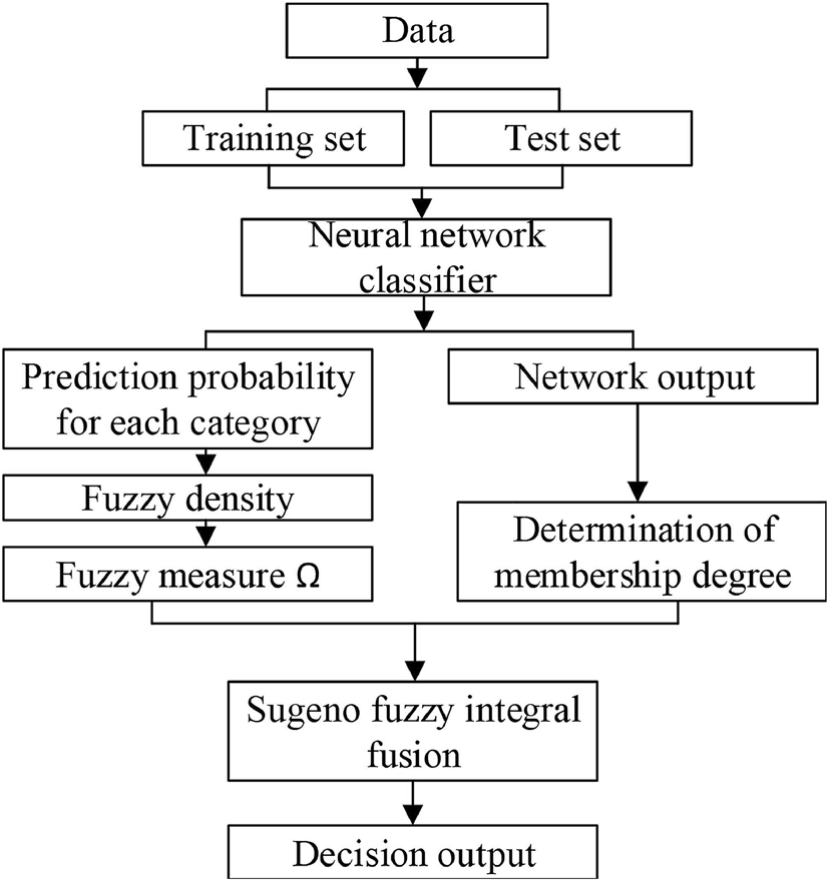
Process of algorithm fusion model.

Sugeno integral is a fuzzy measure integral^41^, the Sugeno fuzzy integral of the function $f:X\rightarrow [0,1]$ on the fuzzy measure $\Omega$ is shown in the expression (6).

6 $$\begin{aligned} \int f(x)d\Omega =\max _{1\le i \le n } (min(f(x_{i}),\Omega (A_{i} ))) \end{aligned}$$

where $\Omega (A_{i} )=(\left\{ x_{i},x_{i+1},...,x_{n} \right\} )$ and $\left\{ f(x_{1}),f(x_{2}),f(x_{3}),...,f(x_{n})\right\}$ is defined as $f(x_{1})\le f(x_{2})\le f(x_{3})\le ... \le f(x_{n})$. The Sugeno fuzzy integral fusion algorithm flow is shown in Fig.7.

## Experimens

### Evaluation index

In the experiment, a variety of criteria are used to evaluate the classification performance of the model, such as confusion matrix, ROC curve and AUC region, accuracy, precision, recall rate and F1 value. Confusion matrix can intuitively show the specific situation of classification of the model, and the confusion matrix is shown in Table 1. TP, FP, TN and FN represent true positive, false positive, true negative and false negative respectively.

**Table 1:** Confusion matrix.

Target	Prediction	
	T (True)	F (False)
P (Positive)	TP	FP
N (Negative)	TN	FN

Accuracy refers to the ratio of the number of samples correctly predicted by the classification model to the total number of samples, which is used to measure the accuracy of the overall prediction of the model. Precision refers to the ratio of the number of samples that are actually positive to the total number of samples that are predicted to be positive. Recall refers to the ratio of the number of samples correctly predicted by the model to the total number of samples that are actually positive among all samples. The F1 value is a harmonic average of accuracy and recall, which is used to comprehensively evaluate the classification performance of the model. The relevant expressions of each evaluation index are shown in Eqs. (7)–(10).

7 $$\begin{aligned} Accuracy= & {} \frac{TP+TN}{TP+FP+TN+FN} \end{aligned}$$

8 $$\begin{aligned} Precision= & {} \frac{TP}{TP+FP} \end{aligned}$$

9 $$\begin{aligned} Recall= & {} \frac{TP}{TP+FN} \end{aligned}$$

10 $$\begin{aligned} F1-Score= & {} \frac{2\times Precision \times Recall }{Precision+Recall} \end{aligned}$$

### Neuron data set

NeuroMorpho.Org^42^is an open neuromorphologic database that collects and shares neuronal morphological data from different species, different brain regions, and different cell types. It contains morphological data on thousands of neurons and is currently the largest publicly accessible 3D neuronal reconstruction and related neuronal dataset.

The two-dimensional image dataset of neurons in the Neuromorph-RAT dataset made by Zhang Tielin’s team^22^ is used for the experiment, including original neuron images (Img_raw) obtained by web crawler, the neuronal images with Z-jumping issues repaired using the tree toolbox (Img_resample), and the neuronal image data with XY alignment (Img_XYalign). In the experiment, 4-category classification and 12-category classification were carried out respectively. The 4-category classification includes principal cells, interneurons, glial cells, and sensory receptor cells, and 12-category classification consists of 12 subclasses, including six principal cells, three interneurons, two glial cells, and one sensory receptor cell.

### Experimental results and analysis

#### Improved tests of AlexNet, VGG11_bn and ResNet-50

**Figure 8:**
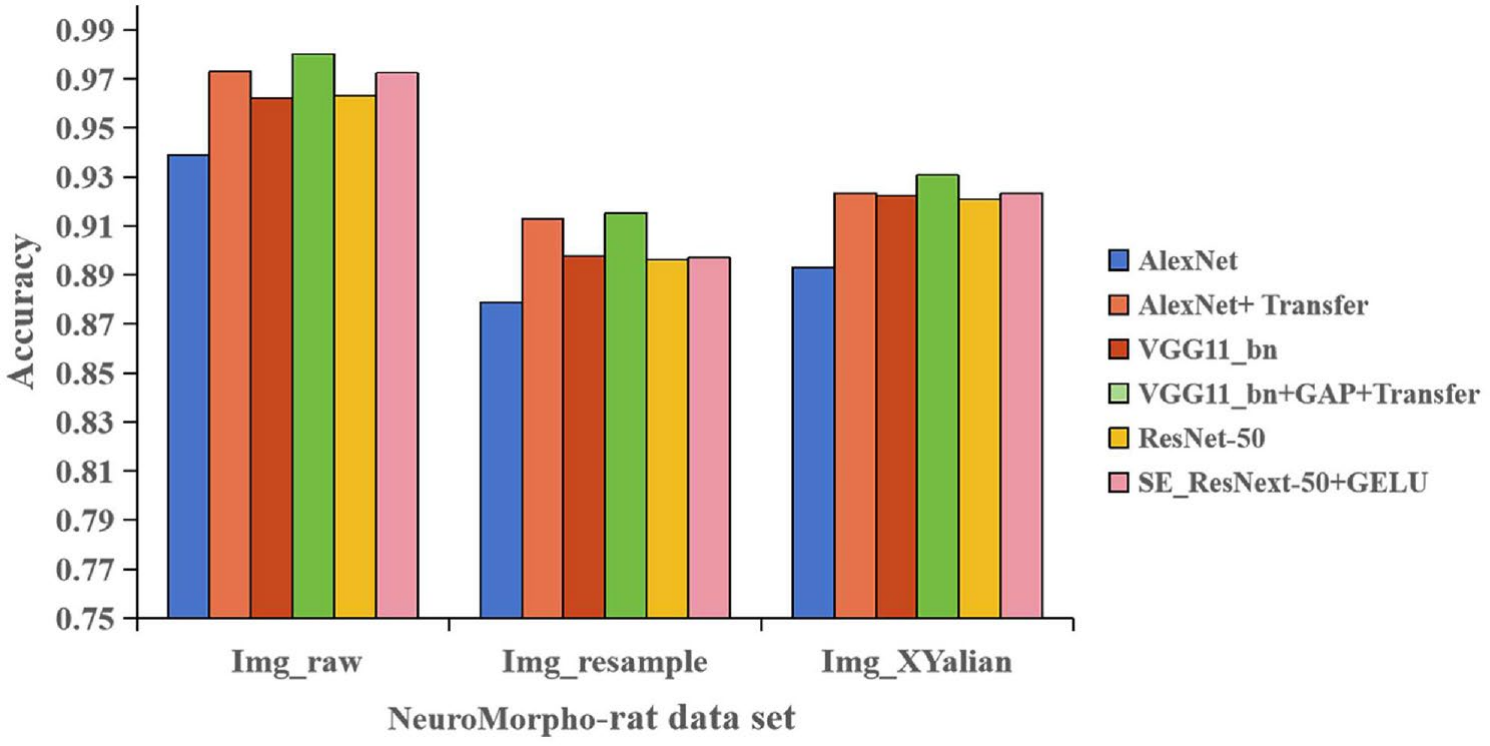
Comparison results of accuracy before and after model improvement (4-category classification).

**Figure 9:**
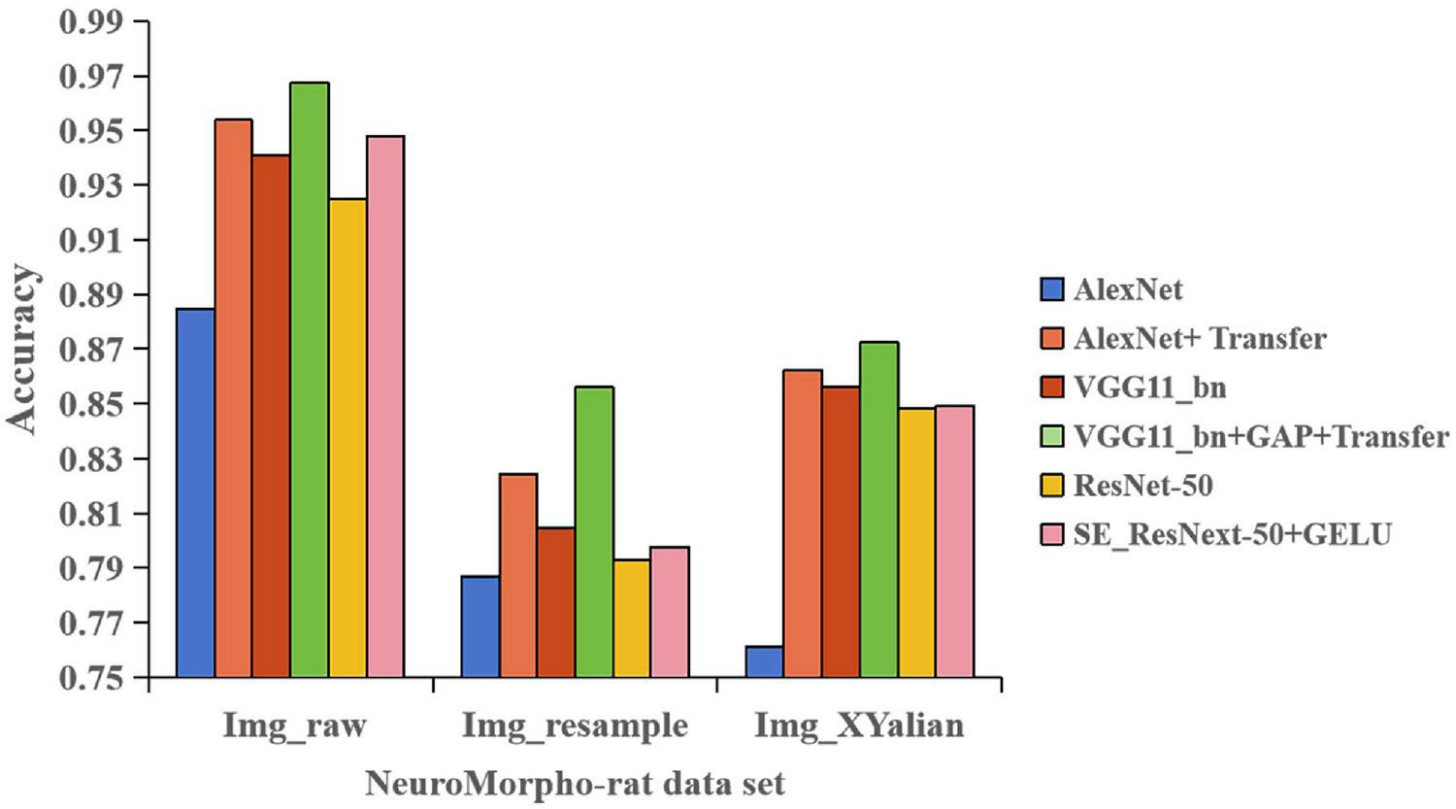
Comparison results of accuracy before and after model improvement (12-category classification).

In order to verify the effectiveness of each improved network, the network model before and after AlexNet improvement, VGG11_bn improvement and ResNet-50 improvement were tested on three datasets under the same experimental conditions. The test results of 4-category classification are shown in Fig. 8. The test results of 12-category classification are shown in Fig. 9.

As can be seen from Fig. 8, the accuracy of the improved AlexNet network, the improved VGG11_bn network and the improved ResNet-50 network were all better than those before the improvement in the neuron morphology classification experiment. In addition, the accuracy of the improved AlexNet has increased by 3.45%, 3.41% and 3.06% compared to the original in the Img_raw dataset, Img_resample dataset, and Img_XYalian dataset, respectively. The accuracy of the improved VGG11_bn has increased by 1.78%, 1.89% and 0.83% on the three datasets, respectively, while the accuracy of the improved ResNet-50 has increased by 0.91%, 0.09% and 0.20% on the three datasets, respectively. This proves that the improved model has better performance in neuronal morphology-4 classification.

As shown in Fig. 9, in the 12-category classification experiment on the three datasets, the improved AlexNet’s accuracy has increased by 6.91%, 3.77%, and 10.09% respectively compared to the original in the Img_raw dataset, Img_resample dataset, and Img_XYalian dataset. The accuracy of the improved VGG11_bn has been improved by 2.63%, 5.13% and 1.60% on the three datasets, respectively, and the accuracy of the improved ResNet-50 has been improved by 2.29%, 0.50% and 0.08% on the three datasets, respectively.

#### Analysis of results of ablation experiment and MCF-Net experiment

Under the same experimental conditions, four different fusion methods of AlexNet network, VGG11_bn network and ResNet-50 network were used for ablation experiments. The experiment also used Img_raw dataset, Img_resample dataset and Img_XYalian dataset. The four fusion schemes include: (1) AlexNet, VGG11_bn and ResNet-50 models before the improvement (2) AlexNet, VGG11_bn before improvement and ResNet-50 after improvement (3) The improved AlexNet, VGG11_bn and ResNet-50 models before the improvement (4) Improved AlexNet, VGG11_bn, and ResNet-50 models. For each fusion method, the following steps are followed to conduct experiments: a. Train three network models to extract features from the images in the datasets. b. Save the probability predicted by the three classifiers for each category in csv file format. c. Use the Sugeno fuzzy integral to fuse the output of each classifier. d. Use the fused features for testing and calculate various experimental indicators. The experimental results are shown in Tables 2, 3 and 4.

By observing the results in Tables 2, 3 and 4, it can be found that the fusion improved model AlexNet, VGG11_bn and ResNet-50, namely MCF-Net model, has the highest classification accuracy in the three datasets, reaching 97.82%, 91.75% and 93.13% respectively. These prove that the proposed fusion scheme of MCF-Net can obtain relatively optimal results, and also demonstrates that the proposed method is effective, and the improvement of a single network can still play its role after the network fusion.

**Table 2:** Test results of different fusion methods in Img_raw dataset.

AlexNet	GAP	Transfer	VGG11_bn	ResNet50	SE	GELU	ACC/%	PER/%	REC/%	F1/%
$\checkmark$			$\checkmark$	$\checkmark$			94.20	89.93	87.20	87.66
$\checkmark$			$\checkmark$	$\checkmark$	$\checkmark$	$\checkmark$	96.14	92.66	91.36	91.22
$\checkmark$	$\checkmark$	$\checkmark$	$\checkmark$	$\checkmark$			97.73	95.62	94.21	94.70
$\checkmark$	$\checkmark$	$\checkmark$	$\checkmark$	$\checkmark$	$\checkmark$	$\checkmark$	97.82	95.75	94.41	94.97

**Table 3:** Test results of different fusion methods in Img_resample dataset.

AlexNet	GAP	Transfer	VGG11_bn	ResNet50	SE	GELU	ACC/%	PER/%	REC/%	F1/%
$\checkmark$			$\checkmark$	$\checkmark$			90.19	85.25	71.61	76.21
$\checkmark$			$\checkmark$	$\checkmark$	$\checkmark$	$\checkmark$	90.30	85.33	71.26	75.93
$\checkmark$	$\checkmark$	$\checkmark$	$\checkmark$	$\checkmark$			91.74	88.45	78.02	82.16
$\checkmark$	$\checkmark$	$\checkmark$	$\checkmark$	$\checkmark$	$\checkmark$	$\checkmark$	91.75	89.05	77.89	82.24

**Table 4:** Test results of different fusion methods in Img_XYalian dataset.

AlexNet	GAP	Transfer	VGG11_bn	ResNet50	SE	GELU	ACC/%	PER/%	REC/%	F1/%
$\checkmark$			$\checkmark$	$\checkmark$			92.57	92.89	77.12	82.72
$\checkmark$			$\checkmark$	$\checkmark$	$\checkmark$	$\checkmark$	92.57	93.47	77.07	82.85
$\checkmark$	$\checkmark$	$\checkmark$	$\checkmark$	$\checkmark$			93.10	93.50	80.00	85.18
$\checkmark$	$\checkmark$	$\checkmark$	$\checkmark$	$\checkmark$	$\checkmark$	$\checkmark$	93.13	94.01	80.10	85.42

**Figure 10:**
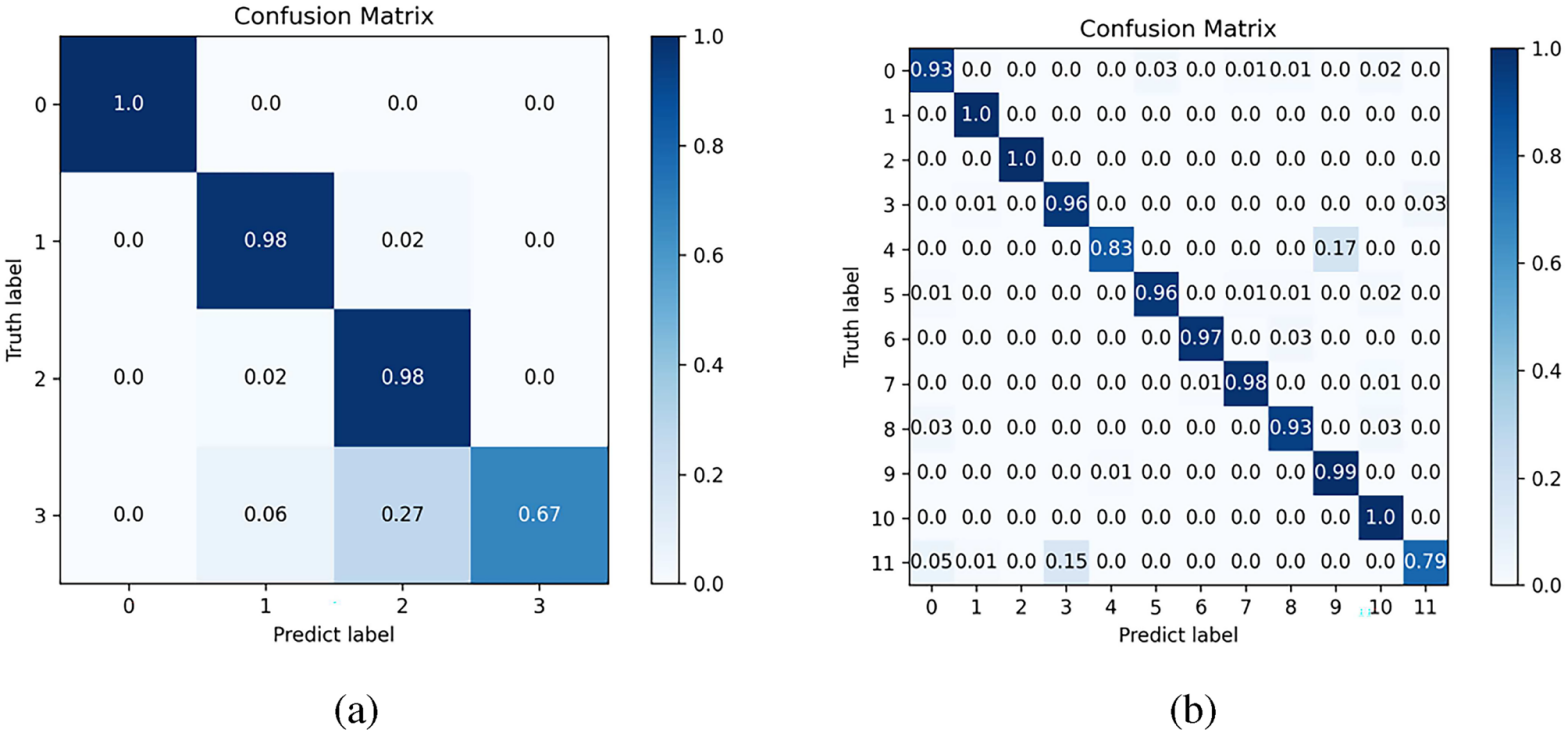
Confusion matrix using MCF-Net on Img_raw dataset (**a**) 4-category classification, (**b**) 12-category classification.

**Figure 11:**
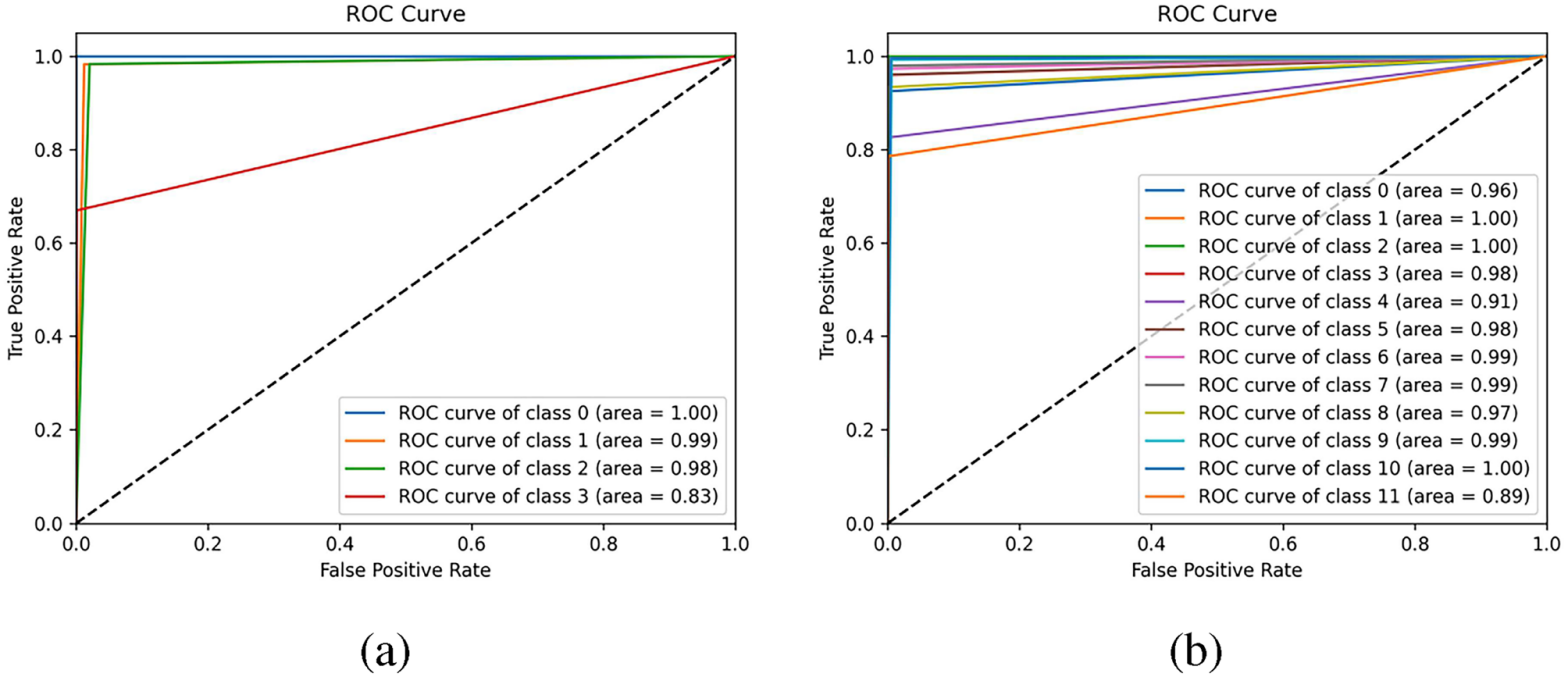
ROC curve using MCF-Net on Img_raw dataset (**a**) 4-category classification, (**b**) 12-category classification.

In order to analyze the classification performance of MCF-Net network, confusion matrix and ROC curve were plotted on Img_raw dataset to compare the classification accuracy of different types of neurons. The confusion matrix is shown in Fig. 10, and the ROC curve obtained under this data set is shown in Fig. 11. To simplify the graph, 0 represents Glia, 1 represents Interneuron, 2 represents Principal cell, and 3 represents Sensory Receptor when drawing the confusion matrix and ROC curve of 4-category classification. When drawing the confusion matrix and ROC curve of 12-category classification, the labels are as follows: 0 represents GABAergic, 1 represents Nitrergic, 2 represents Pakachromaffin, 3 represents Purkinje, 4 represents Astrocyte, 5 represents Basket, 6 represents Ganglion, and 7 represents Granule, 8 represents Medium Spiny, 9 represents Microglia, 10 represents Pyramidal, and 11 represents Sensory Receptor.

In Fig. 10a, it is indicated that when using the Img_raw dataset for 4-category classification testing, except for the slightly lower accuracy of the sensory receptor cell classification, the other categories can be correctly classified. It is evident from Fig. 10b that the MCF-Net model can correctly predict most types of neurons, especially Nitrergic, Pakachromaffin, and Pyramidal cells with an accuracy of 1. The area under the ROC curve (AUC) is a common indicator to measure the performance of a classifier, ranging from 0 to 1, and the closer the curve area is to 1, the better the performance of the classifier. As can be seen from the results in Fig. 11, the area under the ROC curve of the method based on multi-classifier fusion is relatively high, which indicates that the method can classify neurons more accurately.

#### Comparison with existing methods

In order to further verify the validity of the model, we compared the existing classical classification models on three neuronal morphological datasets with 4-category classification and 12-category classification, including the model used for integration in this paper. Zhang Tielin’s team^22^ used CNN(ResNet18) method to classify neuronal image dataset into 12 categories. The comparison results of 4-category classification are shown in Table 5, and the comparison results of 12-category classification are shown in Table 6. According to the experimental results, the MCF-Net network model has a higher accuracy in the task of neuron morphology classification than a single network, which indicates that the network can better capture the characteristics of neuron morphology by combining the classification results of multiple models. In addition, the experimental results of MCF-Net network are verified on three datasets, which further proves the validity and reliability of the network model. In general, the Sugeno fuzzy integral algorithm can be used to fuse the output results of multiple classifiers, which can achieve the morphological classification of neurons well.

**Table 5:** Performance comparison results of different classification methods (4-category classification).

Method	Accuracy(%)		
	Img_raw	Img_resample	Img_XYalign
AlexNet	93.88	87.89	89.63
VGG11	96.22	89.75	92.03
ResNet50	96.32	89.61	92.11
Inception-V3	97.22	90.45	92.09
EfficientNet_B0	97.07	89.93	92.02
Zhang et al.^22^	95.66	83.02	81.98
Proposed	98.04	91.75	93.13

**Table 6:** Performance comparison results of different classification methods (12-category classification).

Method	Accuracy(%)		
	Img_raw	Img_resample	Img_XYalign
AlexNet	88.46	78.68	76.12
VGG11	94.10	80.47	85.63
ResNet50	92.49	79.28	84.91
Inception-V3	92.41	79.64	85.14
EfficientNet_B0	93.99	80.91	84.50
Zhang et al.^22^	86.00	83.24	85.80
Proposed	97.82	85.68	87.60

As shown in Table 7, different integration techniques were used to classify neurons on the Img_raw dataset, including the majority voting rule, the simple average method, and the multiplication rule. The experimental results show that the Sugeno fuzzy integral algorithm has high classification accuracy and good classification performance in neuron classification.

**Table 7:** Comparison with existing integration methods.

Ensemble technique	Accuracy(%)
Majority voting	96.99
Average	97.28
Multiplication rule	97.74
Proposed	97.82

#### The computational complexity analysis

The number of parameters refers to the sum of the number of ownership weight and bias terms in the neural network, which are optimized during the training process to improve the predictive power of the model. The number of parameters depends mainly on the number of filters in the CNN model, the size of each filter, and the number of layers in the network, which together determine the complexity of the model, which in turn has a significant impact on memory requirements and training time. On the other hand, the testing time of a CNN model refers to the time required to predict or classify new and unseen data after the completion of model training, which reflects the response speed and performance of the model in practical applications.

Table 8 presents the detailed parameter information of the three optimized basic networks, including their number of parameters, test time and test accuracy, which are utilized in the Sugeno fuzzy integral integration process. Compared to these three basic networks, the MCF-Net model has a significantly higher number of parameters, almost equal to the sum of the parameters in the three basic networks. The model contains 37.5M parameters and the test time is 252 seconds. As shown in the table, the testing duration of the MCF-Net model includes the individual testing time of the three fundamental networks and the time needed for model integration. Since the model integration time is relatively short, we can roughly consider that the testing time of the MCF-Net model mainly comes from the cumulative testing time of three basic networks. Although the MCF-Net model has a large number of parameters and requires the longest training time, it demonstrates a high accuracy and successfully realizes the goal of neuron classification without considering the limitations of computational cost and experimental conditions.

**Table 8:** Total parameters, test time and test accuracy of the model.

Method	Total parameters(M)	Test time(S)	Test accuracy(%)
AlexNet(Optimized)	2.5	50	95.37
VGG11(Optimized)	9.5	56	96.73
ResNet50(Optimized)	25.5	140	94.78
Proposed	37.5	252	97.82

#### GradCAM analysis

GradCAM^43^is a method for visualizing image regions of concern to convolutional neural network (CNN) models. By combining the convolution layer features and gradient information of the CNN model, it generates gradient-weighted activation maps to highlight the image regions that have an important impact on classification tasks.

**Figure 12:**
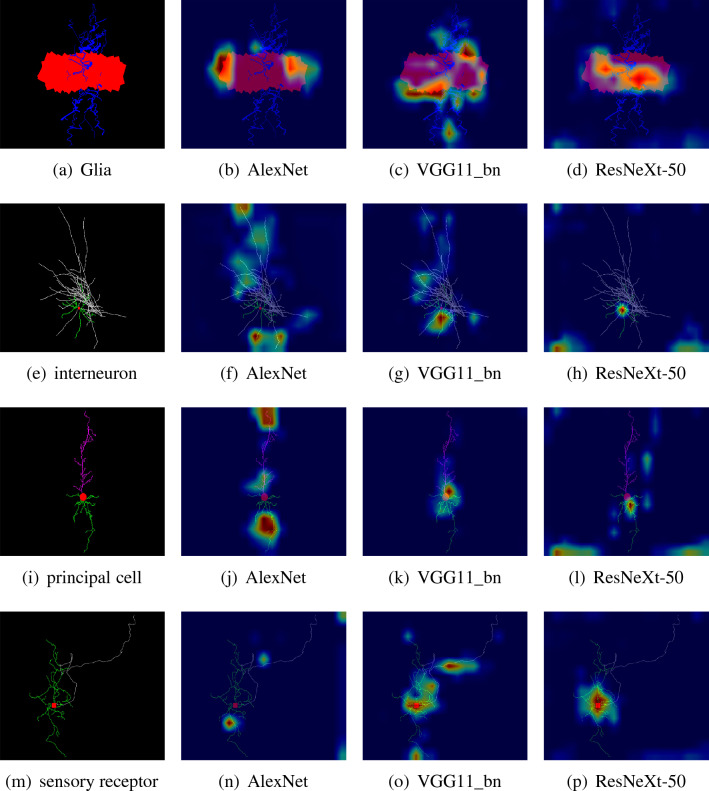
Some neuron images (taken from the Img_raw dataset) along with their GradCAM activations by the three models used for forming the model fusion in this study.

Figure 12 shows GradCAM activation graphs generated by three different models that serve as the basic learning network for ensemble learning, using sample images from the Img_raw dataset. As can be seen from the Fig. 12, different models focus on different parts of the same neuron image. For example, Fig. 12a shows Glia images, and Fig. 12b–d show class activation graphs of AlexNet, VGG11_bn, and ResNeXt-50, respectively. It’s clear from Fig. 12 that AlexNet focuses on both sides of the neuron’s left and right boundaries, and VGG11_bn focused more on the upper right and lower left regions of neurons, while ResNet50 focused on the middle of neurons.

Different models focus on different regions of the same neuron image, which shows that there are differences and complementarities between models, which makes model fusion meaningful. From the examples, it is obvious that the three models focus on different image feature areas. Therefore, the method based on multi-classifiers fusion can synthesize the advantages of multiple models, improve the accuracy and stability of models and achieve better results in practical applications.

## Conclusions

Morphological classification of neurons not only helps to understand the relationship between neuronal structure and function, but also can be used in the diagnosis and research of nervous system diseases. We propose a method to fuse the classification results of multiple classifiers using Sugeno fuzzy integral algorithm. Firstly, the pre-trained model of AlexNet was utilized and its output layer was fine-tuned to reduce training time. Then, in the VGG11_bn network, the traditional fully connected layer was substituted for GAP to reduce the number of parameters, and transfer learning was used to improve the generalization ability of the model. In addition, adding the SE module to ResNeXt-50 enables the network can have feature weights to better extract neuron morphological features. Furthermore, changing the activation function to GELU can improve the model’s ability to fit the data distribution. Finally, the classification results of the three improved networks are fused by using the Sugeno fuzzy integration algorithm. MCF-Net conducted multiple experiments on the three datasets of NeuroMorpho-rat, and the accuracy of 4-category classification was 98.04%, 91.75% and 93.13%, respectively and the accuracy of 12-category classification was 97.82%, 85.68% and 87.60%, respectively. The experimental results show that compared with other commonly used methods, MCF-Net has better performance in the neuron classification test on the three datasets, which can effectively improve the accuracy of neuron morphological classification, and demonstrate the effectiveness of our method. However, our method still has some shortcomings when classifying neurons with similar morphology and structure. In order to solve this problem, more data will continue to be collected for further research.

Although Sugeno fuzzy integration improves the accuracy of neuron morphology classification, it is still difficult to distinguish neuron cells with similar morphology. In order to solve this problem, future studies can focus on classifying the morphology of neurons in three-dimensional space, extracting more comprehensive features using three-dimensional slices, and verifying the generalization of the model. In addition, in order to improve the computational efficiency of neuron morphological classification, integration techniques such as snapshot integration can be considered. At the same time, the combination with other neuroscience research methods, such as electrophysiology and imaging techniques, can also be actively explored to fully understand neuron morphology and function.

## Data Availability

The datasets generated during the current study are available in the https://github.com/thomasaimondy/neuromorpho_neuron_type _classification/tree/master/data_model.

## References

[1] Shepherd GM (2003). The Synaptic Organization of the Brain.

[2] Swanson LW (2012). Brain Architecture: Understanding the Basic Plan.

[3] Markram H (2015). Reconstruction and simulation of neocortical microcircuitry. Cell.

[4] Ascoli GA (2006). Mobilizing the base of neuroscience data: The case of neuronal morphologies. Nat. Rev. Neurosci..

[5] Deitcher Y (2017). Comprehensive morpho-electrotonic analysis shows 2 distinct classes of l2 and l3 pyramidal neurons in human temporal cortex. Cereb. Cortex.

[6] Gillette TA, Ascoli GA (2015). Topological characterization of neuronal arbor morphology via sequence representation: I-motif analysis. BMC Bioinformatics.

[7] Wei Y, He F, Qian Y, Feng F, Wei Y (2023). Neuronal morphology classification based on improved residual network. 2023 IEEE 6th Information Technology, Networking, Electronic and Automation Control Conference (ITNEC).

[8] Ching T (2018). Opportunities and obstacles for deep learning in biology and medicine. J. R. Soc. Interface.

[9] Min S, Lee B, Yoon S (2017). Deep learning in bioinformatics. Brief. Bioinform..

[10] Deb SD, Jha RK (2023). Breast ultrasound image classification using fuzzy-rank-based ensemble network. Biomed. Signal Process. Control.

[11] Kundu R, Singh PK, Mirjalili S, Sarkar R (2021). Covid-19 detection from lung ct-scans using a fuzzy integral-based cnn ensemble. Comput. Biol. Med..

[12] Huang, G., Liu, Z., Van Der Maaten, L. & Weinberger, K. Q. Densely connected convolutional networks. In: *Proc. IEEE conference on computer vision and pattern recognition*, 4700–4708 (2017).

[13] Szegedy, C. *et al.* Going deeper with convolutions. In: *Proc. IEEE conference on computer vision and pattern recognition*, 1–9 (2015).

[14] He K, Zhang X, Ren S, Sun J, He K (2016). Identity mappings in deep residual networks. Computer Vision-ECCV 2016: 14th European Conference, Amsterdam, The Netherlands, October 11–14, 2016, Proceedings, Part IV 14.

[15] Alavi A, Alavi A (2009). Automated classification of dopaminergic neurons in the rodent brain. 2009 International Joint Conference on Neural Networks.

[16] Fengqing H, Jie Z, Fengqing H (2012). Research for neuron classification based on support vector machine. 2012 Third International Conference on Digital Manufacturing & Automation.

[17] Cervantes EP, Comin CH, Junior RMC, Costa LF (2019). Morphological neuron classification based on dendritic tree hierarchy. Neuroinformatics.

[18] Song S, Chen X, Song S, Todo Y (2021). A neuron model with dendrite morphology for classification. Electronics.

[19] Lin X, Zheng J (2019). A neuronal morphology classification approach based on locally cumulative connected deep neural networks. Appl. Sci..

[20] Lin X, Zheng J, Lin X (2018). A 3d neuronal morphology classification approach based on convolutional neural networks. 2018 11th International Symposium on Computational Intelligence and Design (ISCID).

[21] Zheng J, Lin X, Zheng J (2019). Quantitative analysis of influence of morphological feature selection on neuron classification. 2019 11th International Conference on Intelligent Human-Machine Systems and Cybernetics (IHMSC).

[22] Zhang T (2021). Neuron type classification in rat brain based on integrative convolutional and tree-based recurrent neural networks. Sci. Rep..

[23] Ophir O, Shefi O, Lindenbaum O, Ophir O (2023). Neuronal cell type classification using locally sparse networks. 2023 IEEE International Conference on Acoustics, Speech, and Signal Processing Workshops (ICASSPW).

[24] Krizhevsky A, Sutskever I, Hinton GE (2017). Imagenet classification with deep convolutional neural networks. Commun. ACM.

[25] Ioffe, S. & Szegedy, C. Batch normalization: Accelerating deep network training by reducing internal covariate shift. In *International conference on machine learning*, 448–456 (pmlr,) (2015).

[26] Sugeno M, Murofushi T (1987). Pseudo-additive measures and integrals. J. Math. Anal. Appl..

[27] Yang W-J, Xue H-R, Bai J, Yang W-J (2021). Classification and recognition of milk somatic cells based on fusion of fuzzy integral multiple classifiers. 2021 International Conference on Artificial Intelligence and Electromechanical Automation (AIEA).

[28] Lin, M., Chen, Q. & Yan, S. Network in network. Preprint at arXiv:1312.4400 (2013).

[29] Hu, J., Shen, L. & Sun, G. Squeeze-and-excitation networks. In: *Proc. IEEE conference on computer vision and pattern recognition*, 7132–7141 (2018).

[30] Xie, S., Girshick, R., Dollár, P., Tu, Z. & He, K. Aggregated residual transformations for deep neural networks. In: *Proc. IEEE conference on computer vision and pattern recognition*, 1492–1500 (2017).

[31] Hendrycks, D. & Gimpel, K. Gaussian error linear units (gelus). Preprint at arXiv:1606.08415 (2016).

[32] Deng J, Deng J (2009). Imagenet: A large-scale hierarchical image database. 2009 IEEE conference on computer vision and pattern recognition.

[33] Ribani R, Marengoni M, Ribani R (2019). A survey of transfer learning for convolutional neural networks. 2019 32nd SIBGRAPI Conference on Graphics, Patterns and Images Tutorials (SIBGRAPI-T).

[34] Szegedy, C., Vanhoucke, V., Ioffe, S., Shlens, J. & Wojna, Z. Rethinking the inception architecture for computer vision. In: *Proc. IEEE conference on computer vision and pattern recognition*, 2818–2826 (2016).

[35] Wu S-L (2016). Fuzzy integral with particle swarm optimization for a motor-imagery-based brain-computer interface. IEEE Trans. Fuzzy Syst..

[36] Sugeno M (1972). Fuzzy measure and fuzzy integral. Trans. Soc. Instr. Control Eng..

[37] Liu X, Ma L, Mathew J (2009). Machinery fault diagnosis based on fuzzy measure and fuzzy integral data fusion techniques. Mech. Syst. Signal Process..

[38] Keller JM, Liu D, Fogel DB (2016). Fundamentals of Computational Intelligence: Neural Networks, Fuzzy Systems, and Evolutionary Computation.

[39] Tahani H, Keller JM (1990). Information fusion in computer vision using the fuzzy integral. IEEE Trans. Syst. Man Cybern..

[40] Martínez GE, Martínez GE (2016). Comparison between choquet and sugeno integrals as aggregation operators for pattern recognition. 2016 Annual Conference of the North American Fuzzy Information Processing Society (NAFIPS).

[41] Ralescu D, Adams G (1980). The fuzzy integral. J. Math. Anal. Appl..

[42] Ascoli GA, Donohue DE, Halavi M (2007). Neuromorpho. org: A central resource for neuronal morphologies. J. Neurosci..

[43] Selvaraju, R. R. *et al.* Grad-cam: Visual explanations from deep networks via gradient-based localization. In: *Proc. IEEE international conference on computer vision*, 618–626 (2017).

